# Where do we come from? Where are we? Where are we going? President’s speech at the 92nd Annual Meeting of the German Society of Otorhinolaryngology, Head and Neck Surgery on the occasion of its centennial

**DOI:** 10.1007/s00106-021-01137-7

**Published:** 2022-06-01

**Authors:** Stefan K. Plontke

**Affiliations:** Dept. of Otorhinolaryngology, Head and Neck Surgery, Martin Luther University Halle-Wittenberg, Ernst-Grube-Str. 40, 06120 Halle (Saale), Germany


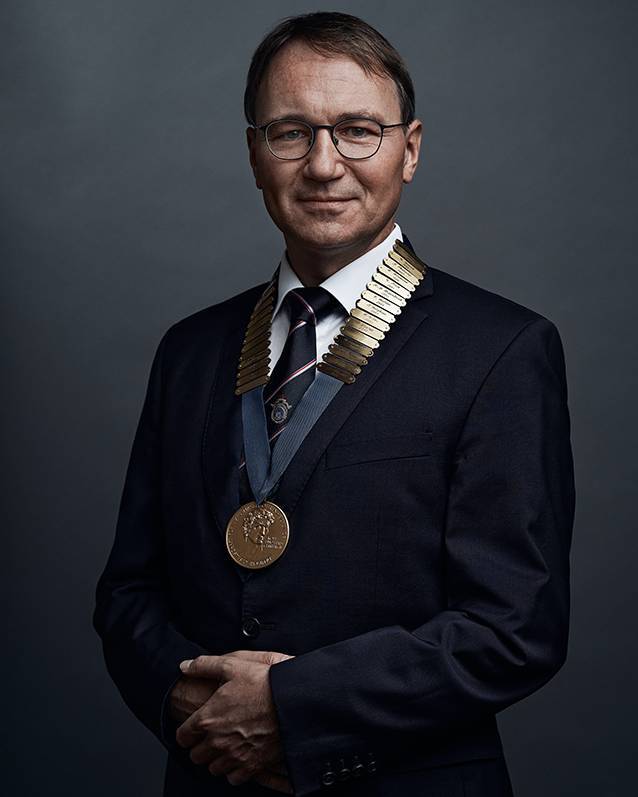


Dear fellow congress president, dear Andreas,

dear honorary presidents, dear Hans-Peter, dear Heinz Maier,

dear members of the executive board of the German Society of Otorhinolaryngology, Head and Neck Surgery,

dear Ellen Lundershausen, vice president of the German Medical Association,

dear Dirk Heinrich, chairman of the German Professional Association of ENT Surgeons,

Spectabilis, dear Professor Gekle,

dear Professor Meller, and

dear colleagues on your screens at home and from wherever you have dialed in:

This month the German Society of Otorhinolaryngology, Head and Neck Surgery (DGHNO-KHC) celebrates a special anniversary. Therefore, I would like to address the questions, ***“Where do we come from? Where are we? Where are we going?”***, which also represent a congress motto.

I would like to preface my remarks with a disclaimer. The American philosopher and writer Ralph Waldo Emerson (1803–1882) noted that we should be aware that our best ideas usually come from others (“Our best thoughts come from others”).

And so, it already begins with the title, chosen in reference to the preface of the main work, *The Principle of Hope*, by the philosopher Ernst Bloch (1885–1977), which he wrote in exile in the United States, and which begins with the words: “Who are we? Where do we come from? Where are we going? What are we waiting for? What awaits us?” [4]. Ernst Bloch is considered a neo-Marxist philosopher—and before questions arise here—I am not a Marxist. The history of Ernst Bloch is interesting because he was called from the United States to the socialist German Democratic Republic (1949–1990) to the chair of philosophy at the (Karl Marx [at that time]) University of Leipzig. However, because he taught his humanist ideas of freedom, e.g., in connection with the Hungarian revolution and the East German uprising of 1953, he was “retired” from the university in 1957 for political reasons and he then emigrated to the Federal Republic of Germany and moved to Tübingen.

There are many quotes that represent why it is important to deal with the past. George Santayana (1863–1952) wrote in *The Life of Reason *in 1905, “Those who cannot remember the past are condemned to repeat it” [38]. And Professor Volker Ladenthin of Bonn wrote in a current issue of the journal *Forschung und Lehre (Research and Teaching):* “We are a presumptuous modernity if we believe everything can always be rethought by each generation” (translated from German [25]).

So first, let us start with the question:

## “Where do we come from?”

The history of otolaryngology can be roughly divided into four phases (Table 1).

**Table 1:** The four phases of the history of otorhinolaryngology

1.	The accumulation and progress of dispersed knowledge by the mid-19th century
2.	The establishment of the first subspecialties of otology, laryngology, and rhinology beginning in the mid-19th century, including their academization: first lectures, specialty consultations and polyclinics, hospitals, associate and later full professorships, journals, books, professional societies, professional congresses, key inventions, and more
3.	The establishment of otorhinolaryngology as a unified specialty in the late 19th and early 20th centuries
4.	The consolidation and further development of otolaryngology with the corresponding recognition of the specialty and its classification in the obligatory subject canon of university teaching, pioneering developments with—from Germany’s point of view—influence on and export to neighboring academic subjects in the 20th century and increasing internationalization

**Fig. 1:**
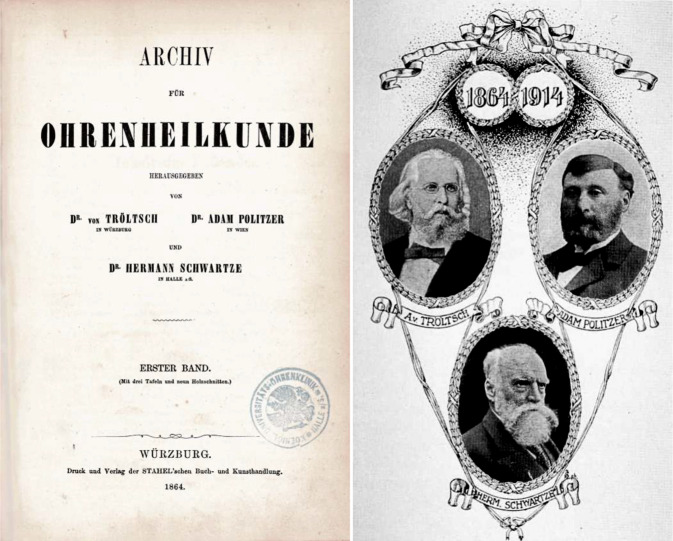
Journals: In 1864, the *Archiv für Ohrenheilkunde *(*Archive of Otology*), the first scientific journal devoted specifically to the (later) field of otorhinolaryngology, was founded by Anton von Tröltsch (1829–1890) from Würzburg, Adam Politzer (1835–1920) from Vienna, and Hermann Schwartze (1837–1910) from Halle (Saale) [31, 34]

**Fig. 2:**
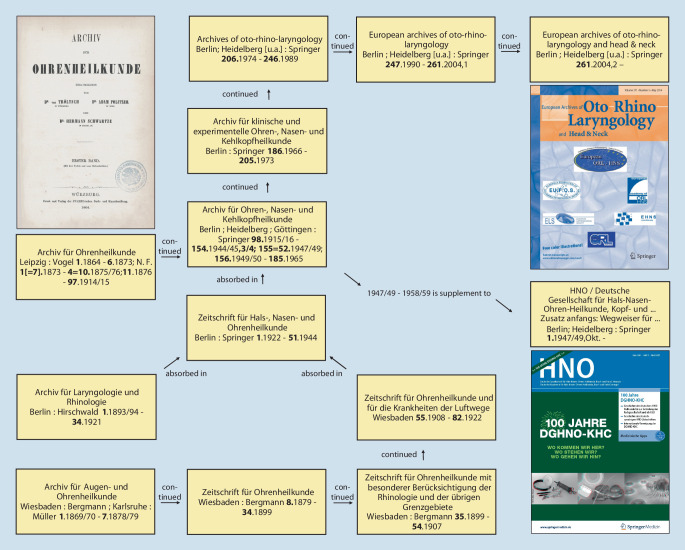
Journals: The *Archiv für Ohrenheilkunde *was renamed* Archiv für Ohren‑, Nasen-, und Kehlkopfheilkunde *(*Archive of Oto-Rhino-Laryngology*) in 1915 and still continues today as the *European Archives of Oto-Rhino-Laryngology and Head & Neck *and as the journal *HNO (ENT)*, which was first published as a supplement [42]

**Fig. 3:**
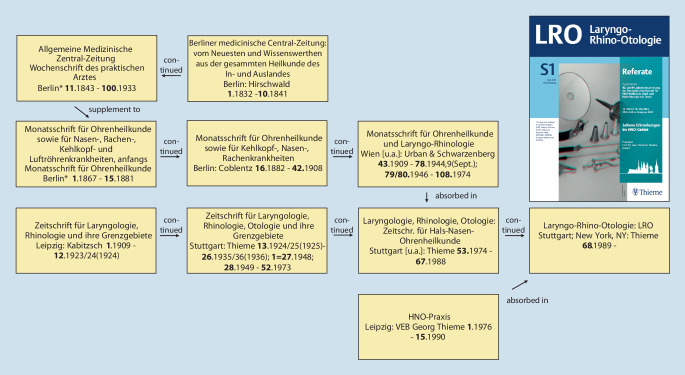
Journals: Today’s journal *Laryngo-Rhino-Otologie *(*Laryngo-Rhino-Otology*) was published from 1867 as *Monatsschrift für Ohrenheilkunde *(*Monthly Journal of Otology*) and from 1882 as *Monatsschrift für Ohrenheilkunde und für Kehlkopf‑, Nasen‑, Rachenkrankheiten *(*Monthly Journal of Otology and Diseases of the Larynx, Nose and Throat*) [42]. Cover picture: with permission © Georg Thieme Verlag KG, all rights reserved

In 1864, the *Archiv für Ohrenheilkunde *(*Archive of Otology*)*, *the first scientific journal worldwide in our later specialty of otorhinolaryngology, head and neck surgery, was founded by Anton von Tröltsch (1829–1890) from Würzburg, Adam Politzer (1835–1920) from Vienna, and Hermann Schwartze (1837–1910) from Halle (Saale) [31, 34]. Today, the journal continues to exist as the *European Archives of Oto-Rhino-Laryngology and Head & Neck *and its former supplement *HNO (ENT)* (Figs. 1 and 2). The journal *Laryngo-Rhino-Otologie *(*Laryngo-Rhino-Otology*) also has old roots: first as *Monatsschrift für Ohrenheilkunde *(*Monthly Journal of Otology*) or *Monatsschrift für Ohrenheilkunde und für Kehlkopf‑, Nasen‑, Rachenkrankheiten *(*Monthly Journal of Otology and Diseases of the Larynx, Nose, and Throat*) as a supplement of the *Allgemeine Medizinische Zentral-Zeitung *(*General Medical Central Gazette*) and later as an independent journal (Fig. 3).

**Fig. 4:**
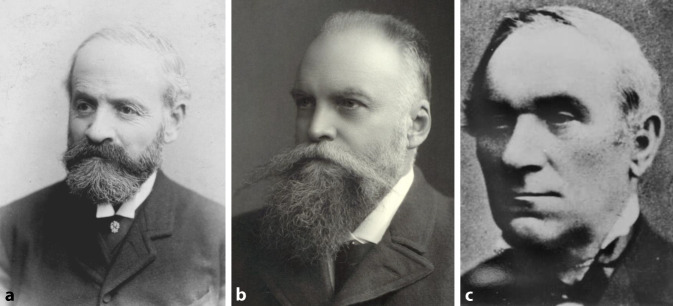
The first German associate professors in the (later) field of otorhinolaryngology. **a** Salomon Moos (1831–1895), 1859 habilitation for internal medicine, 1866 extraordinary professor for otology in Heidelberg. Source: *Graphische Sammlung*, Heidelberg University Library, Graph. Slg. P_1799, Photographer: Eduard Schultze. **b** Hermann Schwartze (1837–1910, 1863 habilitation in otology and 1868 extraordinary professor of otology in Halle (Saale). With permission © University Archives of Martin Luther University Halle-Wittenberg, UAHW, Rep. 40, VI, No. 1, Fig. 39, all rights reserved. **c** Friedrich Rudolf Voltolini (1819–1889), 1860 habilitation and 1868 extraordinary professor of otology *and* laryngology in Breslau. From [51]

About 150 years ago, in the 1860s, Salomon Moos (1831–1895) in Heidelberg, Hermann Schwartze (1837–1910) in Halle (Saale), and Friedrich Voltolini (1819–1889) in Breslau were the first associate professors in our field. It is interesting to note that Moos had first habilitated in internal medicine. Voltolini became associate professor of otology *and* laryngology in 1868 and may thus be considered the first academic representative of the entire field of otorhinolaryngology in Germany (Fig. 4).

**Fig. 5:**
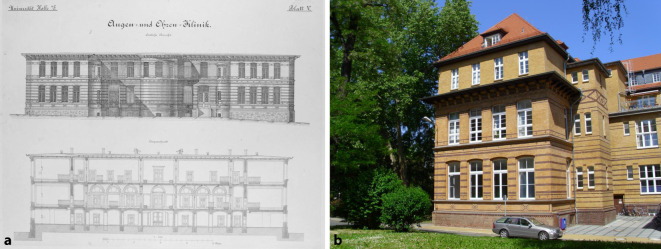
The first hospital building in Germany specifically for the inpatient treatment of patients in our field in 1884: the *Königliche Universitäts-Ohrenklinik *(Royal University Ear Hospital) in Halle (Saale). **a** Construction drawing [8], with permission © University Archives of Martin Luther University Halle-Wittenberg, UAHW, Rep. 8, Folder 91, all rights reserved. **b** Current view of the former men’s ward of the former ear hospital; today, among other things, the dean’s office of the medical faculty. Photo: S. Plontke 2011

In 1884, the *Königliche Universitäts-Ohrenklinik *(Royal University Ear Hospital) was built in Halle (Saale), the first hospital building in Germany specifically for the inpatient treatment of diseases in our field (Fig. 5).

**Fig. 6:**
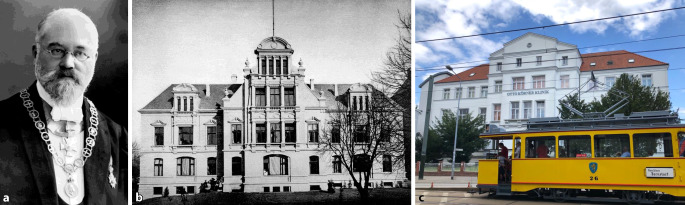
First clinic (1899) and first full professorship (1901) for the entire specialty of otorhinolaryngology in Rostock by Otto Körner. **a** Otto Körner (1858–1935). With permission © Clinic and Polyclinic for Otorhinolaryngology, Head and Neck Surgery “Otto Körner,” Rostock University Medical Center, all rights reserved.** b** The “Grand Ducal University Hospital for Ear, Nose and Throat Patients.” From [52], Rostock University Library. **c** The Otto Körner Clinic today. With permission © R. A. Mlynski, all rights reserved

In 1899, the first university clinic for the entire specialty (otorhinolaryngology) in Germany was opened in Rostock, and Otto Körner was appointed as its director in 1901, becoming the first full professor for the entire specialty of ear, nose, and throat medicine (ENT) in Germany (Fig. 6).

Thus, it has been a long road to the complete academization and establishment of our specialty at universities. For this reason, as well, we should be very critical of the current practices observed at some universities of filling ENT clinic directorships with non-tenured professors, part-time professors, or academic directors outside the field of *otorhinolaryngology, head and neck surgery.*

**Fig. 7:**
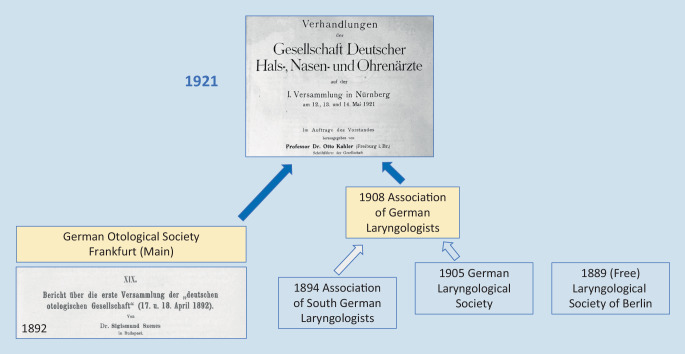
Foundation of the *Gesellschaft Deutscher Hals‑, Nasen-, und Ohrenärzte *(Society of German Otorhinolaryngologists) in 1921 (see text for details)

May 2021 marked a special date for the *German Society of Otorhinolaryngology, Head and Neck Surgery*. Exactly 100 years earlier, at its first annual meeting on May 12–14th, 1921, in the city library in the *Wespennest* (Wasp’s Nest) in Nuremberg, the legal predecessor of our scientific society, the *Gesellschaft Deutscher Hals‑, Nasen-, und Ohrenärzte *(Society of German Otolaryngologists), was formed by the merger of the *Deutsche Otologische Gesellschaft* (German Otological Society) with the *Verein Deutscher Layngologen *(Association of German Laryngologists; Fig. 7).

**Fig. 8:**
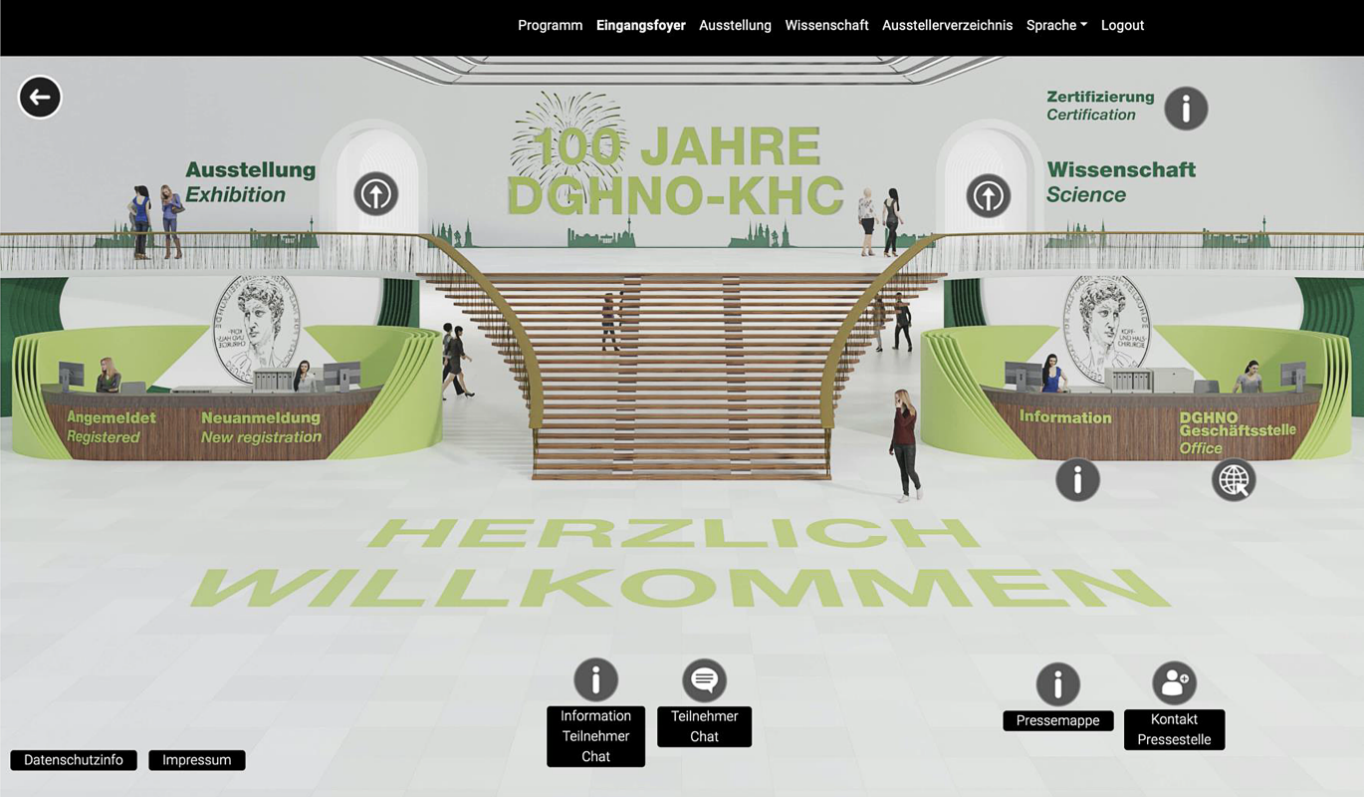
Entrance page of the first complete online annual meeting of the German Society of Otorhinolaryngology, Head and Neck Surgery. With permission © German Society of Otorhinolaryngology, Head and Neck Surgery, all rights reserved. Prepared by VRtual X GmbH (Hamburg, Germany) in collaboration with COCS GmbH (Munich, Germany)

The Wachowskis have Morpheus say in the movie *Matrix*: “Fate, it seems, is not without a sense of irony.” If one considers the COVID-19 pandemic as a stroke of fate, then we must note that for this annual meeting of our society, an “otologist at heart” has joined forces with—in the broadest sense—a “laryngologist at heart.” Exactly 100 years after the founding of our professional society, for the first time in our history, we are presiding as “dual leadership” over this—and here comes another novelty—first completely virtual or online congress of our professional society as joint congress presidents (Fig. 8). We have resolved to do this every 100 years and to submit a corresponding resolution to the executive board. ;‑)

Dear Andreas, I would like to thank you very much for the really good cooperation in this very special year of the COVID-19 pandemic.

At the 20th Annual Meeting in Karlsruhe in 1949, the *Gesellschaft Deutscher Hals‑, Nasen-, und Ohrenärzte *(Society of German Otorhinolaryngologists) was renamed *Deutsche Gesellschaft der Hals‑, Nasen-, und Ohrenärzte *(*German Society of Otolaryngologists*), and at the 39th Annual Meeting in Bad Reichenhall in 1968, it was renamed *Deutsche Gesellschaft für Hals-Nasen-Ohren-Heilkunde, Kopf- und Hals-Chirurgie, e.* *V. Bonn *(German Society of Otolaryngology, Head and Neck Surgery, Bonn).

However, the question, “Where do we come from?” also includes the examination of the role of ENT physicians during the time of National Socialism as well as during the dictatorship in East Germany. In the ENT field, too, there were highly visible scientists who actively collaborated with the National Socialist (Nazi) regime through their activities. Examples are the active cooperation in the Hereditary Health Courts (*Erbgerichtshöfe*) and the scientific work on hereditary hearing loss and deafness that led to guidelines and expert opinions on the basis of which thousands of people were forcibly sterilized and pregnant women forced to have abortions. Thus, ENT specialists were directly involved in the implementation of Nazi racial ideology and bore a substantial share of the responsibility for the suffering of those affected after 1933 [41, 47, 48].

In today’s award ceremonies of our scientific professional society, it is usually said “… shall primarily honor the personality and emphasize the exemplary function of the awardee …”. In 1993, at the 64th annual meeting of our professional society in Münster, the former full professor in Münster, Karl Mündnich, was awarded the honor of the Gold Medal of Merit of the DGHNO-KHC. During the time of National Socialism, Mündnich was *Obersturmbannführer* of the *Leibstandarte Adolf Hitler*, a hand-picked troop, who certainly cannot be characterized as harmless followers [23, 24].

During my research, I was also irritated by the fact that in the 1950s, both in the then-Federal Republic of Germany and in the socialist German Democratic Republic, eyes were obviously firmly closed to the Nazi past in some cases. Woldemar Tonndorf, for example, congratulated Oscar Wagener from Göttingen in an obituary, retrospectively as it were, for a “Jew-free ENT clinic” (“… Wagener had always rejected with sure instinct every employee whose racial affiliation was in the slightest doubt—he had no self-reproaches to make in 1933 …”; translated from German: [44]). In 1951, Tonndorf was appointed as full professor of otorhinolaryngology at the (Karl Marx) University in Leipzig—only a few years before Ernst Bloch (you remember the beginning of this lecture!) lost his teaching license in Leipzig because of free speech.

It is certainly not surprising that in dictatorships, proximity to the state leadership promotes careers; however, we must also think of those whose development as scientists and clinicians was prevented or worse during these times.

**Fig. 9:**
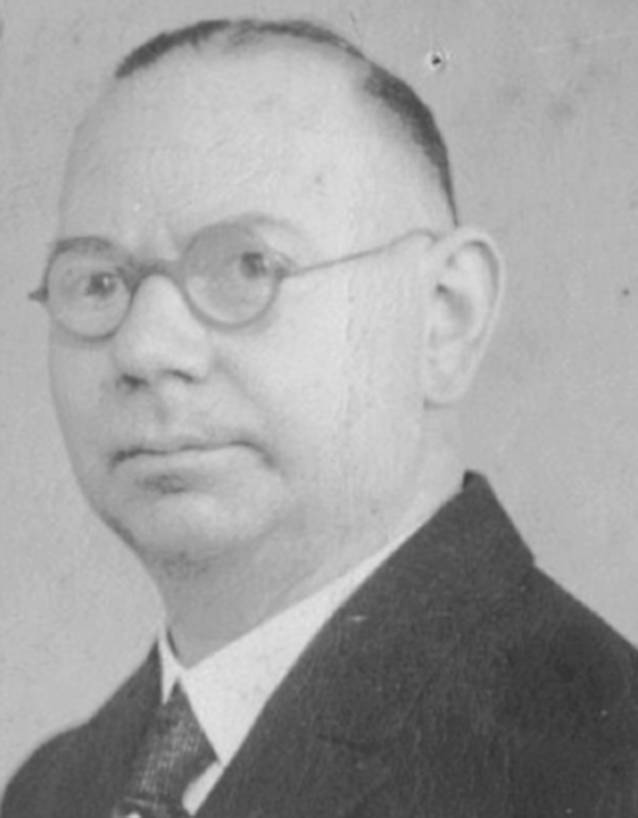
Joseph Cohen (1873–1955) headed the ENT department of the Cologne-Mülheim Municipal Hospital from 1902. When the NSDAP came to power, Cohen was replaced for political reasons. After his license to practice medicine was revoked on September 30th, 1938, Cohen emigrated to the British mandate territory of Tanganyika (now Tanzania) in February 1939, where he worked as a medical practitioner until 1953 [27]

**Fig. 10:**
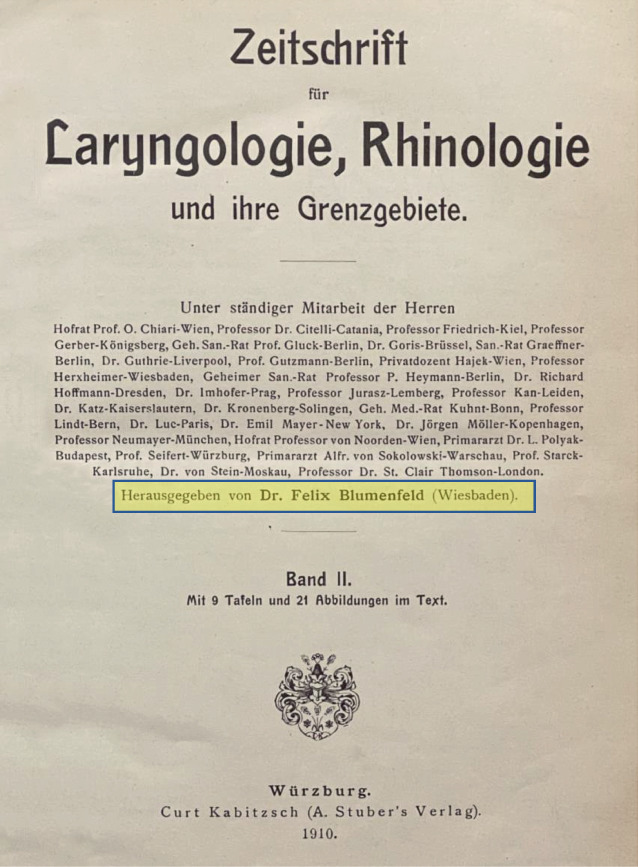
The *Zeitschrift für Laryngologie, Rhinologie, und ihre Grenzgebiete *(*Journal of Laryngology, Rhinology, and* *Their Border Specialties*) was founded in 1909 by the publishing house Curt Kabitzsch in Würzburg. The founding editor was Felix Blumenfeld (1864–1947) from Wiesbaden. His extensive publishing activities have made him well known far beyond the borders of his home region. He was co-editor of several standard works on surgery, pathology, and tuberculosis. Until the National Socialists forced him to withdraw his editorship in 1934, he was continuously editor of this journal (later continued in *Laryngo-Rhino-Otologie, *Thieme-Verlag Stuttgart, see also Fig. 3)

Examples include Josef Cohen (1873–1955), chief physician in Cologne, who was removed from office in 1933 ([27]; Fig. 9), and Felix Blumenfeld (1864–1947) from Wiesbaden, founding editor in 1909 and then editor for many years of the *Zeitschrift für Laryngologie, Rhinologie, und ihre Grenzgebiete *(*Journal of Laryngology, Rhinology, and Their Border Specialties*)—later *Laryngo-Rhino-Otologie*—until his editorship was withdrawn by the National Socialists in 1934 ([39, 42]; Fig. 10).

Although numerous institutions and professional societies in Germany have already addressed their role during National Socialism and have actively reappraised or reprocessed it, there is still a need for action here for our professional society. Therefore, the executive board of the DGHNO-KHC has decided to have the history of German otolaryngology before, during, and after the time of National Socialism scientifically analyzed and reviewed [36].

More about the history can be found in the current issue of the journal *HNO (ENT) *[33], with articles on the German history of otorhinolaryngology up to the foundation of our professional society in 1921 and further from 1921 onward [28, 29], the history of German-language ENT journals [42], and the international networking of our society [32]. I would also like to refer to numerous publications by Harald Feldmann on the history of otorhinolaryngology (e.g., [9–12]) and to the overview article by Karl Heinz Vosteen on the “Development of Otorhinolaryngology in the 19th Century,” which appeared in 1996 in the anniversary volume on the academic teaching institutions and teachers in otorhinolaryngology [46], as well as to the published commemorative lecture of Konrad Fleischer on the occasion of the 75th anniversary of the foundation of the society in 1996 [13].

**Fig. 11:**
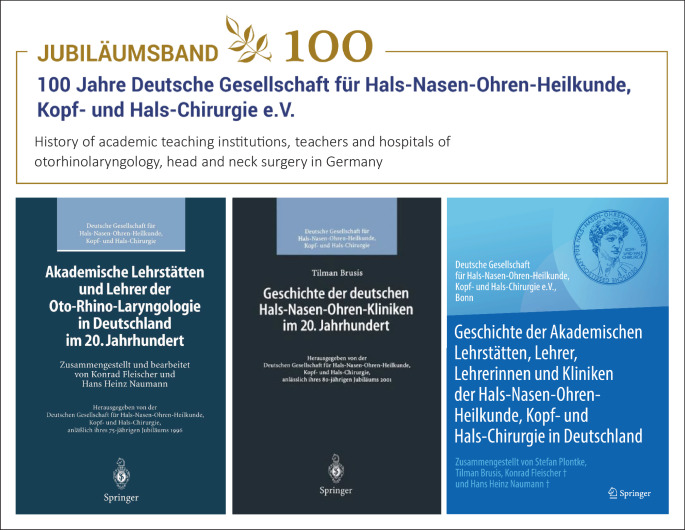
More on the history of German otorhinolaryngology also can be found in the book edited on the occasion of the 100th anniversary year of the foundation of our professional society*, Geschichte der akademischen Lehrstätten, Lehrer und Lehrerinnen und Kliniken der Hals-Nasen-Ohren-Heilkunde, Kopf- und Hals-Chirurgie in Deutschland *(*History of Academic Teaching Institutions, Teachers and Hospitals of Otorhinolaryngology, Head and Neck Surgery in Germany*)*.* It will unite its two predecessors published in 1996 and 2001 into one volume and update the history of the university ENT hospitals in the last 25 years and that of the non-university ENT hospitals in the last 20 years [1]

A supplement will be the book, *Geschichte der akademischen Lehrstätten, Lehrer und Lehrerinnen und Kliniken der Hals-Nasen-Ohren-Heilkunde, Kopf- und Hals-Chirurgie in Deutschland *(*History of Academic Teaching Institutions, Teachers and Hospitals of Otorhinolaryngology, Head and Neck Surgery in Germany*), which is planned for the anniversary year of our professional society. It will unite its two predecessors published in 1996 and 2001 into one volume and will update the history of university ENT hospitals in the last 25 years and that of non-university ENT hospitals in the last 20 years. Many developments in German otolaryngology from its institutional beginnings to the year 2021 can also be found in the chapters on the respective ENT hospitals ([1]; Fig. 11).

**Fig. 12:**
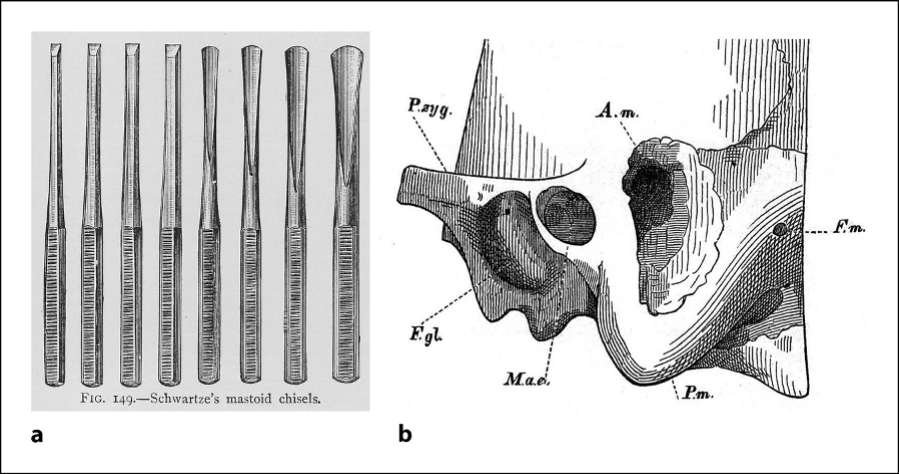
Surgical opening of the mastoid process with chisels. **a** Mastoid chisels according to Schwartze. From Dench’s Textbook, 1896 [7]. **b** Schematic drawing of the opening of the mastoid process from the original publication by Schwartze and Eysell in 1873 [40]

At this point, I will take the liberty of allowing myself a little local patriotism. I would like to look back to the chisel, which was certainly beneficial when it was introduced into ear surgery by Hermann Schwartze in Halle (Saale) (Fig. 12). Alfred Schulz van Treeck from Berlin described in 1951 in the *Textbook for ENT Medicine *that the chisel also has its risks: “As a precaution, the facial muscles innervated by the facial nerve are observed by an assistant during this act [ear/mastoid surgery with chisel]. Twitching indicates that the facial nerve is in danger; sometimes, however, the observed twitch is the last because it already indicates transection” (translated from German).

That leads me to the second part of my presidential address and the question:

## “Where are we?”

Here, a small selection of medical advances in otolaryngology are mentioned first (Table 2).

**Table 2:** Selection of medical advances in otolaryngology

Techniques of tympanoplasty with titanium middle ear prostheses
Implantable electronic hearing systems and successes in inner ear surgery
Hearing rehabilitation with cochlear implants for deafness and partial deafness
Defect reconstruction with microvascular anastomosed grafts
Precise, intensity-modulated radiotherapy or heavy ion radiotherapy
Chemotherapy
Modern immunotherapies
Surgical assistance systems (“surgical robots”)
Intraoperative imaging and navigation
Breakthrough advances in imaging, histological and molecular genetic diagnostics

The second question, “Where are we?,” is the focus of our anniversary congress, which, in addition to some historical aspects with the lectures, symposia, roundtable discussions, and “Oxford-style” debates, as well as the involvement of industry partners, is dedicated to the main scientific topics of “Quality in Medicine” (as the topic of the 2020 annual meeting, which was canceled because of the COVID 19 pandemic) and “Rare Diseases in Otorhinolaryngology” [35].

However, answering the question, “Where are we?,” also involves looking beyond our own borders, and here we must note that there are significant differences in access to healthcare around the world [3, 5, 21].

Moreover, global malnutrition currently leads to more disease and death than do violence, use of drugs, alcohol and tobacco, and unprotected sex combined [49]. Of the 56 million deaths worldwide in 2012, a total of 620,000 (about 1%) were caused by violence (including 120,000 from war and 500,000 from crime), 800,000 from suicide, and about 1.5 million from diabetes. One could also summarize it as, “sugar is now more dangerous than gunpowder” (quoted from [17]).

This leads into the third part of my speech, and thus the third question:

## “Where are we going?”

Stephen Hawking (1942–2018) in his posthumously published book *Brief Answers to the Big Questions* [18] summarized the really big questions we should be asking ourselves (Table 3).

**Table 3:** The really big questions

1.	Is there a God?
2.	How did it all begin?
3.	Is there other intelligent life in the universe?
4.	Can we predict the future?
5.	What is inside a Black Hole?
6.	Is time travel possible?
7.	Will we survive on Earth?
8.	Should we colonize space?
9.	Will artificial intelligence outsmart us?
10.	What does the future hold?

Cited from: Stephen Hawking, *Brief Answers to the Big Questions* [18]

As a scientific society, we are likely to deal with very few of these issues.

Specifically, however, we face challenges in the following areas in the coming years (Table 4):

**Table 4:** Challenges for ENT in the coming years (focusing on German ENT)

1.	Specialization versus unity of the field
2.	Digitalization, “big data,” and artificial intelligence
3.	Economization/commercialization
4.	Prioritization and resource allocation (linked to the ethical issues that arise from this and that are particularly evident, e.g., in the topic of *rare diseases*, but also in the context of the current COVID-19 pandemic)
5.	Increase in outpatient medicine and surgery
6.	Increase in bureaucracy
7.	Feminization (in terms of proportion among medical staff) and increasing interest in working part-time
8.	Academization/de-academization
9.	Industrialization and economization of research
10.	Personalization of medicine
11.	Mechanization—surgical assistance systems/robotics
12.	Paradigm shift: increasing proportion of drug therapies and gene therapy
13.	Education and training

Even 100 years after the foundation of our professional society, we are committed to the ***unity of the field of otorhinolaryngology, head and neck surgery***. Nevertheless, the breadth of the specialty, the multitude of (rare) diseases, the rapid medical–technical progress, the economic framework conditions with simultaneous quality demands in diagnostics and therapy, as well as the surgical expertise inherently required in a surgical specialty and based on surgical experience and “practice” result in the ***necessity of specialization*** even within our specialty. Future medical care structures must accommodate a broad provision of basic outpatient and inpatient ENT specialty care in all regions in Germany, as well as specialized, technical, interdisciplinary, and interprofessional care at centers. This specialization is also necessary to enable protection of the entire range of our specialty by the greatest possible knowledge, abilities, and skills against “covetousness” of other specialties, while at the same time supporting interdisciplinary cooperation for the benefit of the patients.

***Specialty ENT training*** must meet the high demands of the very broad yet very specialized knowledge in our specialty. It must not be minimized and must be based on solid financing models—in the inpatient as well as in the outpatient areas. I believe that extensive or even complete specialty training (“residency”) in otorhinolaryngology in purely outpatient care structures is the wrong way to go. Experience in the field of ophthalmology in Germany has already shown us the consequences of this undesirable development. If further training is outsourced from the large hospital training centers to private ENT practices, then this reduces the training experience of generations of trainees. That cannot be good for the patients who need our help, and, last but not least, we ourselves may be patients later on.

Closely related to the necessary specialization within our field is ***preclinical and clinical research***. In this context, it is important to address current problems in research and research funding. A high proportion of biomedical studies have methodological deficiencies, most commonly poor experimental design (“underpowered”), inappropriate or poor statistics, selective reporting of data, and publication bias toward positive results. Often the study design is not at all adequate to answer a particular question. John Ioannidis of Stanford University even came to the conclusion in 2005 that “most published research findings are false” and too many results are not reproducible [20]. Science and science funding have become too much of a business, with the evaluation of scientific success being problematic. As a false incentive, quantity (e.g., amount of funding and numbers of publications) has developed instead of quality and creativity.

The funding of a small number of universities under the German government’s Excellence Initiative “[…] contends with a classic organizational problem. The incentive structure of the initiative is often interpreted as rewarding primarily the quantity of scientific output. Quality falls by the wayside. […] The number of publications per professorship has increased at all German universities, at non-excellence universities even more than at excellence universities. But over the same period, the average number of citations per publication fell—and particularly sharply in the group of universities of excellence […]” (translated from: [19]).

The question of the importance of the size of universities and research groups is also controversial and must be viewed in a differentiated manner. A group of researchers from Chicago and Evanston in Illinois evaluated 65 million articles, patents, and software products that span the period 1954–2014. They demonstrated “that across this period, smaller teams have tended to disrupt science and technology with new ideas and opportunities, whereas larger teams have tended to develop existing ones.” The group concluded that “both small and large teams are essential to a flourishing ecology of science and technology,” and suggested that “to achieve this, science policies should aim to support a diversity of team sizes” (cited from [50]). This is especially important because “the German Research Foundation systematically favors the larger institutions. […] Oligarchization in the university landscape, however, fails to exploit scientific potential” (cited from: [15]). Just like individual hospitals, research groups and research associations can lose their “resonance frequency” once they reach a certain size.

With ***The German Study Centre for Otorhinolaryngology, Head and Neck Surgery (DSZ-HNO)***, an important step has been taken toward strengthening evidence-based medicine in ENT and the clinical study culture in our field. However, economic pressure, among other things, hinders the promotion of young scientific and medical talent at universities. As a professional society, we must actively counteract de-academization, promote clinician scientists, and counteract the orientation toward education and training according to a non-academic “physician school” model. Specialization tends to mean more academization in ENT.

Structurally, the increase in outpatient medicine and surgery will be with us as an important change in the coming years. We will have to ensure that technically complex operations are performed under outpatient conditions to the same quality standards as currently in the inpatient setting and that they are also adequately compensated [6]. This development is not compatible with the current financing systems, including mixed calculation systems with flat rates per case in the inpatient sector. It is also quite unclear or even questionable whether a higher proportion of outpatient ENT surgeries will be the answer to and the solution for the nursing shortage in Germany. The equation “outpatient = low effort = cheap” is certainly wrong. In any case, medical care across inpatient and outpatient healthcare sectors will become increasingly important in the treatment of ENT diseases.

With respect to ***digitization and artificial intelligence***, it is also important to remember Alan Turing (1912–1954), who laid much of the theoretical groundwork for modern information and computing technology, and Konrad Zuse (1919–1995), the designer of the world’s first working computer. “The lives and health of people in Germany could be better protected if the possibilities of digitization in healthcare were used responsibly and in a scientifically sensible way.” This is the conclusion reached by the German Council of Health Experts in its current report, which was presented to Minister Spahn in March 2021 (translated from press release to [14]). “The goal should be the reorientation of healthcare: towards a digital, a systematically learning healthcare system.” In the process, health data should not fall into the wrong hands. At the same time, it must be gotten into the right hands. The Council of Health Experts recommends a strategy for digitizing the healthcare system. This includes, for example, (a) framework conditions for digitization, including trade-offs in the area of data protection; (b) recommendations on electronic patient records; and (c) recommendations on data use for more targeted research, prevention, diagnostics, and therapy [14]. In the field of artificial intelligence and machine learning, there are also efforts and activities in the ENT area. A review paper on the topic identified 90 studies that investigated the use of artificial intelligence, particularly in the areas of image analysis, speech analysis, gene analysis, and electroencephalography and electrocardiography (for the Apnea–Hypopnea Index) using clinical data, and reported initial successes while also postulating a need for further research [43].

“Digitization, artificial intelligence, and big data are being touted as a concept that puts us on the threshold of precision medicine that will soon diagnose and treat without error. With the right technical upgrades to master the vast amounts of data, the end of chance and thus error-free medicine are already waiting on the horizon. These are the promises. A closer look, however, reveals disillusionment. Of the usual three dimensions of technology assessment—namely, benefit, risk, and cost—the last two are ignored as much as possible, and the assessment is reduced to a promise of benefit, which is usually completely exaggerated. Most important, even with the greatest faith in technology, the impact on humanity in medicine cannot be overlooked” (translated from [2]).

Our efforts—absolutely also at the political level—must be directed against the waste of resources through ***increasing bureaucratization***, inappropriate ***economization***, and the expansion of ***false incentives.***

In this context, close cooperation with physicians from other specialties and within our field between the scientific society and the German Professional Association of ENT Surgeons (BV-HNO) is absolutely essential, despite different convictions or diverging interests in some aspects and factual issues.

An economic course of action is also important in healthcare. But we must decide: Do we want a ***healthcare system or a healthcare market***? Is healthcare a matter of public interest or should it be regulated by the market? If we think that the market will take care of it, then we must realize that the market serves itself first and foremost. The state, however, has a responsibility for the lives and health of its citizens. State-guaranteed revenues from a system financed on the basis of solidarity should not be a source for lucrative profit objectives. In the ENT field, we must create a nationwide existential care for the population at a high level, including by realizing different levels of care, instead of directing patients through “advertising,” “portal practices,” or similar with the purpose of maximizing profits. We must also question our own role as “employees” in the system: Are we “free health professionals” or simple “vicarious agents” for the generation of profits for stock corporations, foreign pension funds, or other financially strong outside investors [16, 26, 30, 37, 45]?

**Fig. 13:**
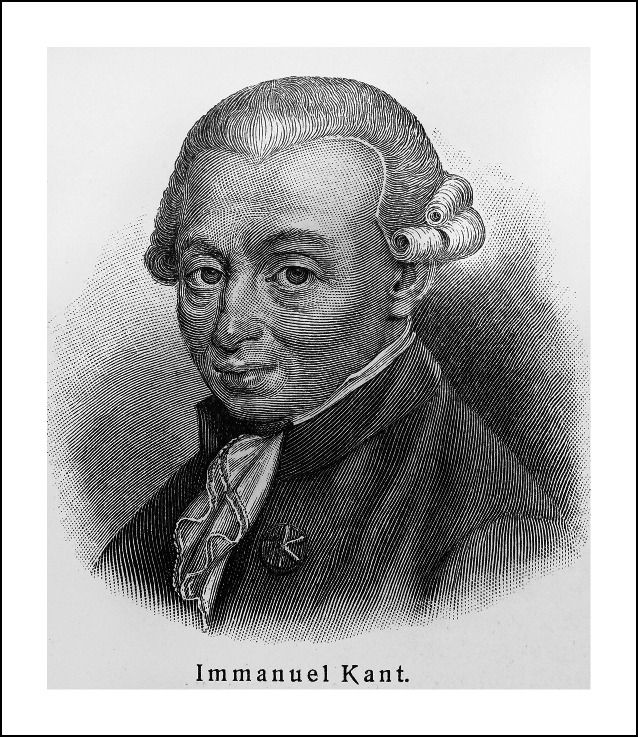
Immanuel Kant (1724–1804): “Self-purpose formula” as a prohibition of instrumentalization. With permission © nickolae/stock.adobe.com, all rights reserved

To the question, ***“Are patients a means to an end or an end in themselves?,”*** a look at history again gives a clear answer. In Kant’s “self-purpose formula,” the prohibition of instrumentalization of other people and thus of patients is anchored: “Act in such a way that you treat humanity, whether in your own person or in the person of any other, never merely as a means to an end, but always at the same time as an end” ([22]; Fig. 13).

We must maintain or regain the medical responsibility for the care process to a large extent, so that in the end—completely in the Kantian sense—the human being always remains the purpose of our actions.

Thank you for lending me your ears!

Stefan K. Plontke

2020/2021 President of the German Society of Otorhinolaryngology, Head and Neck Surgery

## Declarations

For this article no studies with human participants or animals were performed by any of the authors. All studies mentioned were in accordance with the ethical standards indicated in each case.

The supplement containing this article is not sponsored by industry.
